# Racial and ethnic differences in the associations between social integration, C-reactive protein and depressive symptoms

**DOI:** 10.1016/j.ssmph.2020.100663

**Published:** 2020-09-04

**Authors:** Alexander Chapman, Alexis R. Santos-Lozada

**Affiliations:** a Department of Sociology and Criminology, The Pennsylvania State University, USA; b Population Research Institute, The Pennsylvania State University, USA; c Department of Human Development and Family Studies, The Pennsylvania State University, USA

**Keywords:** Social integration, NHANES, Depressive symptoms, Population health, Inflammation, Race/ethnicity

## Abstract

This study evaluates whether the associations between social integration, inflammation, and depressive symptoms vary by race/ethnicity in the United States. Our study includes 5,634 respondents age 40 and older from the National Health and Nutrition Examination Survey for 2005-2008. We fit multivariate logistic regression models with interactions between C-reactive protein (CRP) and race/ethnicity as well as social integration and race/ethnicity to test our hypotheses. We find that social integration and CRP operate independently in their associations with depressive symptoms by race/ethnicity. Higher levels of social integration are associated with lower predicted probability of depressive symptoms for White and Black populations. This association is not statistically significant for the Hispanic population. CRP is associated with depressive symptoms for the White population, but not the Black or Hispanic populations. Our results suggest that studying depressive symptoms, and other mental health outcomes, among the US population without considering variation by race/ethnicity may restrict scholarly understanding of health disparities. Population-based assessments of associations between physiological processes or social integration should consider whether these variables operate differently by race/ethnicity and work to explain why differences may emerge. Furthermore, interventions aimed at social integration may improve mental health among older adults in the United States; especially for the least socially integrated.

## Highlights

• Social integration and inflammation operate independently in their associations with depressive symptoms.
• Associations between mental health, inflammation, and social integration differ by race/ethnicity.
• Inflammation is associated with depressive symptoms for non-Hispanic White but not for non-Hispanic Black or Hispanic populations.
• Social integration protects against depressive symptoms for non-Hispanic White and Black populations but not for the Hispanic population.
• Approaches to health disparities research should consider variations by subpopulations.

## 1 Introduction

In 2017, 17.3 million adults suffered from a major depressive episode in the United States, accounting for 7.1% of the adult population (NIMH, 2019). The diagnostic criteria for a major depressive episode are met when a person experiences at least five of nine symptoms which must include either depressed mood or loss of interest or pleasure. These symptoms must cause clinically significant distress unrelated to drugs or another medical conditions (APA, 2013). In turn, depressive symptoms are associated with adverse health outcomes such as suicide, but also increased risk of stroke (Pan et al., 2011). Research shows that non-Hispanic Black and Hispanic populations experience more depressive symptoms relative to the non-Hispanic White^1^ population (Abrams & Mehta, 2019; Musliner et al., 2016). Given the consequences of depressive symptoms and health disparities experienced by marginalized racial/ethnic groups, it is important to study their determinants and how they may vary by race/ethnicity among middle-age and older adults.

Depressive symptoms are caused by the complex interplay of social and physiological processes. Separate bodies of research implicate both social integration/isolation and chronic inflammation as predictors of depressive symptoms. Evidence suggests that elevated levels of inflammation lead to depressive symptoms (Smith et al., 2018) while social integration is inversely associated with depressive symptoms (Kiecolt-Glaser et al., 2010). Some research finds that inflammation mediates the association between social integration and health (Uchino et al., 2018). However, this association varies by subpopulations such as race/ethnicity (Nersesian et al., 2018). Despite this scholarly work, studies that examine the relationship between social integration, inflammation, and depressive symptoms have mixed findings and have not yet considered variation by race/ethnicity.

In this study, we use multivariate logistic regression to test whether inflammation and social integration are differentially associated with depressive symptoms by race/ethnicity in the United States. We pool two waves of the National Health and Nutrition Examination Survey (NHANES), 2005–2006 and 2007–2008. These data allow us to generalize our findings to non-institutionalized middle-age and older adults in the United States. Our study contributes to research on racial/ethnic health disparities and suggests that social and physiological predictors of depressive symptoms vary by race/ethnicity in the United States.

## 2 Background

### 2.1 Racial/ethnic differences in inflammation and depressive symptoms

A well-established body of research finds an association between inflammation and depressive symptoms (Howren et al., 2009; Smith et al., 2018; Valkanova et al., 2013). Inflammation is often measured with biomarkers such as C-Reactive Protein (CRP). Biomarkers are advantageous because they provide an objective measure of health and wellbeing. Physiological explanations propose that elevated levels of CRP may lead to depressive symptoms because of how cytokines, which induce synthesis of proteins like CRP, interact with neurotransmitter metabolism, neuroendocrine function, and neural plasticity (Dantzer et al., 2008; Miller et al., 2009; Miller & Raison, 2016). A meta-analysis of longitudinal research that focuses on the association between CRP and depressive symptoms finds that elevated levels of CRP predict depressive symptoms (Valkanova et al., 2013). Another meta-analysis of thirty-two studies, which focuses on exclusively older adult samples in both cross-sectional and longitudinal research, finds that inflammation leads to depressive symptoms (Smith et al., 2018).

Black and Hispanic populations have higher levels of CRP compared to the White population (Nazmi & Victora, 2007; Santos-Lozada & Daw, 2018), which may explain disparities in depressive symptoms. However, the association between CRP and depressive symptoms varies by race/ethnicity, and the results are mixed (Case & Stewart, 2014; Deverts et al., 2010; Stewart, 2016). Case and Stewart (2014) highlight that elevated levels of CRP are associated with depressive symptoms for White respondents, but they find no association for Black or Hispanic respondents. By contrast, Deverts et al. (2010) find that elevated levels of CRP are associated with depressive symptoms for Black but not for White respondents. Some explanations of why racial/ethnic marginalized groups have higher levels of inflammation focus on factors such as discrimination (Stepanikova et al., 2017), weathering (Geronimus et al., 2006), and disparities in income level and educational attainment (Howard & Sparks, 2016; Stepanikova et al., 2017). However, despite the negative association between social integration and support and inflammation (Kiecolt-Glaser et al., 2010; Yang et al., 2013), researchers have yet to thoroughly consider social integration as an explanation of racial/ethnic variation in inflammation levels.

### 2.2 Racial/ethnic differences in social integration and depressive symptoms

A well-established body of literature emphasizes the protective effects of social relationships^2^ against depressive symptoms for different sectors of the population (Gariepy et al., 2016; Sonnenberg et al., 2013). One way to measure social relationships is through social integration. Social integration is the amount or frequency of social interaction (Gottlieb & Bergen, 2010; Kiecolt-Glaser et al., 2010). Researchers measure social integration with several components such as participation in religious organizations, marital partnership, number of close friends, and participation in volunteer organizations or clubs (Ford et al., 2006; Rees et al., 2010). Social integration is dynamic, differing throughout the life course and by socioeconomic status (Ertel et al., 2008). For example, older adults rely more on spouses and have a stronger protective affect from close friends than younger populations (Gariepy et al., 2016).

While research finds that social integration reduces depressive symptoms, its positive influence is unequal by race/ethnicity (T.-C. Yang & Park, 2019) and specifically in its association with depressive symptoms (Almeida et al., 2011). This may be partly due to the types of integration and sources of social support (T.-C. Yang & Park, 2019). Black respondents report smaller, tighter social networks where family members comprise a greater share than White respondents (Ajrouch et al., 2001). Research also indicates that familism or “*familismo*”, defined as strong identification and attachment to nuclear and extended families, is more attributable to Hispanic populations’ social structure than other racial/ethnic groups (Desmond & Turley, 2009). As a result of this unique characteristic, those of Hispanic origin often hold strong feelings of loyalty, reciprocity, and solidarity within their families (Sabogal et al., 1987) and have higher levels of familial support relative to the White population (Almeida et al., 2009). Additionally, family support may be more protective against mental illness than other sources of support (Almeida et al., 2011). Religious attendance varies by race/ethnicity where the Black population is more likely to be religiously involved than other groups (Brown et al., 2015). Social integration may influence racial/ethnic groups differently given values such as familism, religious attendance, or friendship composition.

### 2.3 Social integration, inflammation, and depressive symptoms

A meta-analysis finds that social integration is negatively associated with inflammation and that inflammation is an important link between social integration and health (Uchino et al., 2018). Arguments posed in the literature suggest that social integration influences levels of CRP through health behaviors and conditions such as diet, physical activity, and psychosocial stress (Ford et al., 2006; Kiecolt-Glaser et al., 2010). These conditions and behaviors cause the body to respond with inflammation. However, findings are not always consistent when examining subpopulations. The association between social integration and inflammation differs by gender (Y. C. Yang et al., 2013), age (Ford et al., 2006), and race (Nersesian et al., 2018).

Despite established dyadic links between social integration, inflammation, and depression, the association between all three remains ambiguous. Hur et al. (2018) find that social support buffers the relationship between CRP and depressive symptoms among men, but not women. Among female cancer patients, baseline feelings of social support are associated with fewer depressive symptoms and lower levels of inflammation at the end of the study (Hughes et al., 2014). In a sample of 316 adolescents, Guan et al. (2016) find that friendship support does not buffer the relationship between depressive symptoms and CRP, but parental support does; the authors do not find significant associations by race/ethnicity or gender. Lastly, a clinical study of young adults (n = 39) finds that inducing an inflammation response leads to feelings of social disconnection and depressed mood, but also that accounting for social disconnection attenuates the relationship between the inflammation challenge and depressed mood (Eisenberger et al., 2010).

### 2.4 Present study

The purpose of this study is to test whether CRP levels and social integration are associated with depressive symptoms and how those associations may differ by race/ethnicity in the United States. Some studies test associations between social ties, inflammation, and depressive symptoms, but among these, the only study to even control for race/ethnicity is among adolescents (Guan et al., 2016). Other studies do not consider variations by race/ethnicity likely due to lack of sample variation (Hur et al., 2018) or sample size (Eisenberger et al., 2010). Additionally, this emerging area of research focuses on social support rather than social integration, and results may change with a different metric of social ties.

We propose three hypotheses. First, there will be a positive association between elevated levels of CRP, above a clinically determined threshold, and depressive symptoms; but the relationship may vary by race/ethnicity even when controlling for potential covariates. In other words, we expect that association between CRP and depressive symptoms is moderated by race/ethnicity in line with prior research (Case & Stewart, 2014; Deverts et al., 2010). Our second hypothesis is that higher levels of social integration will be associated with fewer depressive symptoms but that the association varies by race/ethnicity, while controlling for potential covariates. Our third hypothesis is that after incorporating both social integration and CRP, the association between CRP and depressive symptoms will be attenuated; conditional on a relationship between CRP and depressive symptoms. However, we expect this finding to vary by race/ethnicity. Research indicates that after accounting for feelings of social disconnection, the relationship between inflammation and depressive symptoms is no longer statistically significant (Eisenberger et al., 2010).

## 3 Data and methods

### 3.1 Analytic sample

We use NHANES 2005–2008 data for our analysis. The NHANES is a nationally representative survey conducted by the National Center for Health Statistics that uses a stratified, multistage probabilistic sampling strategy to provide national estimates of health pertaining to the civilian, non-institutionalized population of the United States (Johnson et al., 2013). These data are collected in repeated cross-sections of unique respondents. For this paper, we use three sections of the NHANES: The Demographic Section which covers demographic and socioeconomic characteristics for each respondent, the Laboratory Section provides a CRP measure, and the Questionnaire Section has variables to measure depressive symptoms and social integration. We combine these sections for two consecutive waves from 2005 to 2008 to create a pooled cross-sectional dataset. Though NHANES has repeated cross-sections from 1999 to 2020, we limit our study to 2005–2008 because the survey questions about social integration were discontinued in 2008.

6797 participants ages 40 and older completed the demographic interview and Medical Examination Component (MEC) of the NHANES from 2005 to 2008. To create our final dataset, we removed participants with missing data on any variable. We exclude 376 respondents that have a missing value for CRP, including eight respondents with a CRP level of 10 mg/dL or higher because it is indicative of an infection (Nehring et al., 2020). We remove 462 respondents who do not answer the depression screener. Next, we remove seven respondents that do not answer the alcohol questionnaire and three that do not answer the smoking questionnaire. We also remove seven and nine respondents that have missing information related to diabetes or hypertension respectively. We exclude 81 respondents due to missing BMI, including an outlier; a respondent with a BMI more than double the next closest respondent. Finally, we exclude respondents with missing educational attainment (n = 4) and respondents with missing information on any of the social integration measures (n = 45). Among the remaining 5803 respondents, 169 identify as other race/ethnicity. We performed two-tailed t-tests among respondents with a missing information for the depression screener, CRP, social integration, and those of other race/ethnicity. These tests were performed by race/ethnicity when testing for mean differences on the depression screener, CRP, and social integration. We find that mean differences are not significant among White, Black, or Hispanic respondents (results not shown). However, other race/ethnicity respondents may be different than other groups by CRP and social integration (results not shown). We omit other race/ethnicity respondents from the analysis because of sample size and because we cannot make comparisons across racial/ethnic groups with this category. Our final analytic sample includes 3128 White, 1177 Black, and 1329 Hispanic respondents.

### 3.2 Measures

#### 3.2.1 Depressive symptoms

We measure depressive symptoms using the Patient Health Questionnaire (PHQ)^3^ included in the NHANES Questionnaire Data. We use the first 8 items of the PHQ.^4^ Researchers often count depressive symptoms by summing the answers on the PHQ. Scores of the PHQ-8 can range from 0 to 24 because each question ranges from 0 (“Not at all”) to 3 (“Nearly every day”). Research affirms the validity of using a score of 10 as the threshold on the PHQ-8 (Kroenke et al., 2009; Razykov et al., 2012). Thus, we dichotomize this measure by whether the respondent scores at least 10 on the PHQ-8.

#### 3.2.2 Race/ethnicity

The Demographic Section of NHANES provides the source variable for race/ethnicity which includes five categories: Mexican American, other Hispanic, non-Hispanic White, non-Hispanic Black, and other race (including multi-racial respondents). We combine the categories of Mexican Americans and other Hispanics into a single variable that includes all Hispanic respondents. We exclude respondents that identify as other race as noted previously. Thus, we measure race/ethnicity as a categorical variable: White, Black, and Hispanic.

#### 3.2.3 C-reactive protein (CRP)

We use CRP levels that examiners obtain during the MEC of the NHANES. The NHANES measures CRP continuously as mg/dL. Scholarship notes that the clinically determined threshold of CRP ≥0.30 mg/dL poses a high risk for adverse health consequences (Howard & Sparks, 2016). We dichotomize CRP to indicate whether the CRP level of each respondent is above or below the clinical threshold.

#### 3.2.4 Social Network Index

We obtain social network information from the NHANES Social Support Module. The module only includes data from adults age 40 and older. The social support questions originate from the Social Network Index-Alameda County Study (Berkman & Syme, 1979). Researchers use these questions to calculate the Social Network Index (SNI) (Ford et al., 2006; Loucks et al., 2006). SNI indicates the level of social integration through four types of social connections: marital status (married or not), sociability (number of close friends or relatives^5^), church membership, and other community organization membership.

We follow the SNI scoring algorithm of previous research with slight modifications. While the original SNI studies have a range from zero to four, the NHANES survey does not have information on membership in community organizations. Therefore, we score the SNI, similar to Rees et al. (2010), by adding three components: married (No = 0 and Yes = 1), participation in religious services (once a month or more = 1, otherwise = 0), and sociability (four or more close friends/relatives = 1, otherwise = 0). The survey design does not allow respondents to identify close friends and relatives separately. Our SNI ranges from zero to three, where higher values indicate greater social integration.

#### 3.2.5 Demographic and socioeconomic Characteristics, Health behaviors and conditions

We incorporate the following covariates in our regressions: age, sex, education, smoking habits, drinking habits, hypertension, diabetes, and body mass index (BMI). These covariates have well-established associations between the outcome and the independent variables of interest (see Ford et al., 2006; Nehring et al., 2020; Yang et al., 2014). We measure age categorically: 40–54 years (reference group), 55–64 years, 65–74 years, and 75 years and over. We operationalize sex as a dichotomous variable. We measure educational attainment categorically as less than high school, high school or some college, and four-year college degree or higher. We measure smoking behavior categorically as non-smoker, current smoker, and former smoker. We measure alcohol consumption with three categories—non-drinker, drinker, and heavy drinker—given its J-shaped relationship with CRP (O'Connor & Irwin, 2010). We include those that drink more than the median amount (36 days yearly) in the heavy drinker category and include all other drinkers (1–36 days yearly) in the drinker category. We account for hypertension and diabetes with dichotomous measures for each, based on the respondents' report of ever having a diagnosis. We create a dichotomous measure of BMI where greater than 25.0 indicates overweight/obese.

### 3.3 Analytic strategy

We conduct all analyses using STATA 15 (Stata, 2017). We account for complex survey design and weighting of the observations with NHANES survey weights where our sampling unit, stratum, and weights are SDMVPSU, SDMSTRA, and WTMEC4YR respectively (Lee & Forthofer, 2006). We use the NHANES sample design and weights to account for the unequal probability of selection and to adjust standard errors which make results generalizable to the non-institutionalized White, Black, and Hispanic population in the United States ages 40 and older.

**Fig. 1:**
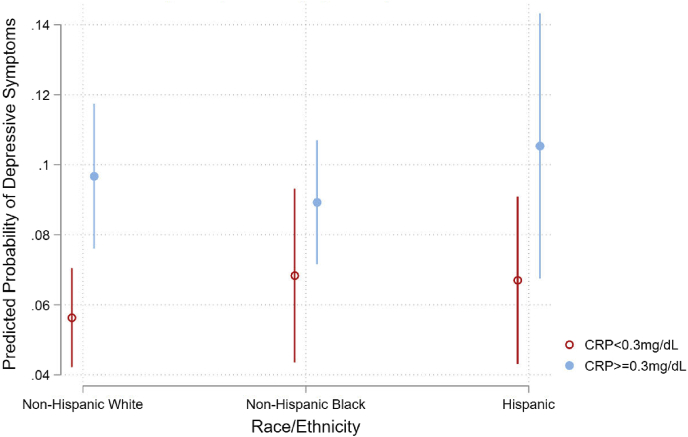
Predicted probability of depressive symptoms by C-reactive protein and race/ethnicity.

**Fig. 2:**
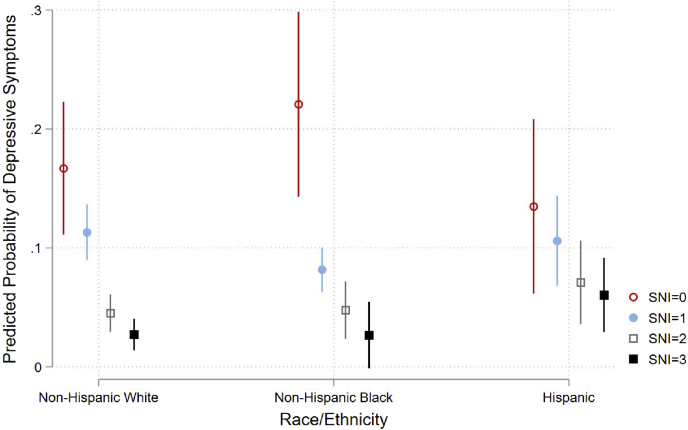
Predicted probability of depressive symptoms by social integration and race/ethnicity.

We provide weighted descriptive statistics overall and by race/ethnicity (Table 1). After the descriptive results we test our hypotheses using nested multivariate logistic regression (Table 2). We show Fig. 1 and Fig. 2 to plot the predicted probabilities from the interaction models shown in Table 2. We do not interpret the coefficients or standard errors from the interaction terms because those terms are not necessarily indicative of statistical significance in nonlinear models (see Ai & Norton, 2003; Mize, 2019). To simplify this, we plot the predicted probabilities (Figs. 1 and 2) and test for statistical significance following Mize (2019) in the Supplemental File (Tables S4 and S5). The Supplemental File also includes a correlation matrix of all variables (Table S1), analyses with the PHQ-9 as the dependent variable (Table S2), full models similar to Table 2 that exclude either CRP or SNI (Table S3), and interactions between SNI and CRP (Table S3). We present all results as odds ratios (OR) with corresponding standard errors and significance levels.

**Table 1:** Weighted descriptive statistics for Depressive Symptoms, CRP, SNI, Sociodemographic Characteristics and Health Behaviors for U.S. Adults 40 and Older by Race/Ethnicity, NHANES 2005–2008.

	Overall	NH White	NH Black	Hispanic
	Proportion	Proportion	Proportion	Proportion
Depressive Symptoms	0.07	0.07_c_	0.10	0.10_a_
Elevated CRP	0.38	0.36_b_	0.48_ac_	0.42_b_
Social Network Index				
SNI = 0	0.10	0.09_b_	0.12_ac_	0.10_b_
SNI = 1	0.29	0.27_bc_	0.37_a_	0.33_a_
SNI = 2	0.39	0.39_b_	0.35_ac_	0.41_b_
SNI = 3	0.23	0.24_bc_	0.16_a_	0.16_a_
Age Group				
40–54 years	0.51	0.49_bc_	0.56_ac_	0.63_ab_
55–64 years	0.22	0.22_bc_	0.24_a_	0.20_a_
65–74 years	0.15	0.16_c_	0.13_c_	0.11_ab_
75 years and older	0.12	0.13_bc_	0.06_a_	0.05_a_
Gender				
Male	0.47	0.47	0.43	0.49
Female	0.53	0.53	0.57	0.51
Educational Attainment				
Less than High School	0.18	0.14_bc_	0.29_ac_	0.48_ab_
High School Grad	0.54	0.56_bc_	0.52_ac_	0.39_ab_
College Grad	0.27	0.30_bc_	0.19_ac_	0.12_ab_
Smoking				
Non-smoker	0.49	0.47_bc_	0.52_ac_	0.56_ab_
Current smoker	0.20	0.19_bc_	0.26_ac_	0.17_ab_
Former smoker	0.31	0.33_bc_	0.23_a_	0.26_a_
Drinking Habits				
Non-drinker	0.22	0.21_b_	0.29_ac_	0.23_b_
Drinker	0.33	0.33_bc_	0.29_ac_	0.36_ab_
Heavy Drinker	0.46	0.47_bc_	0.41_a_	0.41_a_
Diabetes	0.11	0.10_bc_	0.20_ac_	0.17_ab_
Hypertension	0.42	0.42_bc_	0.54_ac_	0.32_ab_
Overweight/Obese	0.73	0.72_bc_	0.78_ac_	0.80_ab_
Unweighted N	5634	3128	1177	1329

Note: Subscript characters represent a statistically significant difference (p < 0.05) between subgroups: a = White, b = Black, and c = Hispanic. Elevated CRP indicates a C-reactive protein level above the clinically determined threshold of 3.0 mg/dL.

**Table 2:** Weighted logistic regression predicting depressive symptoms among U.S. Adults 40 and older, NHANES 2005–2008 (n = 5634).

	Model 1		Model 2		Model 3		Model 4		Model 5		Model 6	
	OR	SE	OR	SE	OR	SE	OR	SE	OR	SE	OR	SE
Elevated CRP	1.674***	(0.215)			1.592***	(0.196)	1.272†	(0.167)	1.240†	(0.157)	1.305†	(0.202)
Social Network Index												
SNI = 1			0.577**	(0.093)	0.578**	(0.093)	0.601**	(0.108)	0.659†	(0.151)	0.599**	(0.106)
SNI = 2			0.247***	(0.044)	0.252***	(0.045)	0.315***	(0.059)	0.301***	(0.065)	0.314***	(0.059)
SNI = 3			0.152***	(0.033)	0.156***	(0.034)	0.225***	(0.054)	0.210***	(0.068)	0.224***	(0.054)
Race/Ethnicity												
Black	1.391†	(0.251)	1.282	(0.197)	1.221	(0.194)	0.944	(0.149)	1.453	(0.474)	1.092	(0.265)
Hispanic	1.508*	(0.255)	1.454*	(0.256)	1.428†	(0.248)	1.196	(0.193)	0.765	(0.320)	1.245	(0.266)
Age Group												
Age 55-64							0.648***	(0.074)	0.648***	(0.074)	0.652***	(0.073)
Age 65-74							0.417***	(0.070)	0.421***	(0.072)	0.419***	(0.071)
Age 75+							0.463***	(0.091)	0.476***	(0.091)	0.470***	(0.090)
Male							0.629**	(0.079)	0.618**	(0.081)	0.622**	(0.082)
Educational mAttainment												
HS Graduate							0.755	(0.143)	0.757	(0.142)	0.752	(0.142)
College Graduate							0.366**	(0.104)	0.373**	(0.103)	0.369**	(0.104)
Health Behaviors												
Current Smoker							1.942**	(0.359)	1.984***	(0.351)	1.979***	(0.348)
Former Smoker							1.240	(0.229)	1.245	(0.232)	1.241	(0.230)
Drinker							0.931	(0.099)	0.927	(0.094)	0.925	(0.095)
Heavy Drinker							0.670**	(0.079)	0.672**	(0.080)	0.677**	(0.082)
Health Conditions												
Diabetes							1.522*	(0.288)	1.504*	(0.297)	1.509*	(0.294)
Hypertension							1.730**	(0.327)	1.758**	(0.338)	1.754**	(0.334)
Overweight/Obese							1.169	(0.207)	1.131	(0.200)	1.129	(0.198)
Interactions												
Black x SNI = 1									0.471*	(0.160)		
Hispanic x SNI = 1									1.207	(0.525)		
Black x SNI = 2									0.730	(0.287)		
Hispanic x SNI = 2									2.148	(1.142)		
Black x SNI = 3									0.673	(0.483)		
Hispanic x SNI = 3									3.055†	(1.721)		
Black x CRP											0.755	(0.209)
Hispanic x CRP											0.908	(0.292)
Constant	0.058***	(0.009)	0.193***	(0.033)	0.157***	(0.031)	0.243***	(0.064)	0.220***	(0.068)	0.221***	(0.064)

Note: Elevated CRP indicates a C-reactive protein level above the clinically determined threshold of 3.0 mg/dL. The reference group for SNI is zero. The reference group for race/ethnicity is White. Reference group for age group is 40–54 and is less than high school for educational attainment. The reference group for health behaviors and conditions are no behavior or no condition.

Standard errors in parentheses. †p < 0.1, *p < 0.05, **p < 0.01, ***p < 0.001.

## 4 Results

In the descriptive results (Table 1), we include subscript letters that mark significant differences between each racial/ethnic group. A subscript “a”, “b”, or “c” indicates statistically significant differences from White, Black, or Hispanic populations respectively. Responses to the PHQ suggest that 7% of White, 10% of Black, and 10% of Hispanic populations meet the depressive symptom threshold with a significant difference between White and Hispanic populations. The White population meets the clinical CRP threshold in 36% of cases, significantly lower than the Black population (48%), but not the Hispanic population (42%). The White population has higher levels of social integration compared to the other populations, with a significantly higher score compared to the Black population when SNI equals 2, and higher than both the Hispanic and Black populations when SNI equals 3. With respect to demographic characteristics, the Hispanic population is younger and less well educated than both the White and Black populations. Females make up a disproportionate share of the population for each racial/ethnic group; this is expected as women live longer than men on average, and our analytical sample consists of middle-age and older adults. Among the health behaviors, the Hispanic population has the smallest proportion of current smokers at 17%, followed by 19%, and 26% for White and Black populations. About 47% of the White population drinks alcohol more than 36 times yearly compared to 41% for both Black and Hispanic populations. The Black population is disproportionately diagnosed with health conditions compared to White and Hispanic populations. Lastly, the Black and Hispanic populations are more commonly overweight or obese than the White population. These descriptive statistics suggest that the Black and Hispanic populations generally have worse health and fewer resources to improve or maintain their health than the White population.

In Table 2 we provide the odds ratios from logistic regression models predicting depressive symptoms. In Model 1, we estimate depressive symptoms with clinical CRP threshold and race/ethnicity. An elevated level of CRP is associated with 67% greater probability of depressive symptoms. The estimates for Black and Hispanic populations are higher than White, though marginally significant for the Black population. In Model 2, we estimate SNI and race/ethnicity rather than CRP. These estimates suggest that higher levels of SNI decrease the probability of depressive symptoms. In this model the estimates are not significantly different for the Black relative to White population, but they are significantly higher for the Hispanic population.

Model 3 includes CRP and SNI in the same model. These estimates are similar to previous models. However, the Hispanic-White difference is marginally significant and the point estimate for CRP decreases slightly relative to Model 1, but the coefficient remains statistically significant. An elevated level of CRP is associated with a 58% increased probability of depressive symptoms. This supplies little evidence that SNI attenuates the relationship between CRP and depressive symptoms. In Model 4 we add control variables to Model 3. The relationship between CRP and depressive symptoms is now marginally statistically significant, and the race/ethnicity predictors are not significantly different. Older populations have lower probabilities of depressive symptoms relative to the 40-54-year-old population. A four-year college degree decreases the probability of depressive symptoms relative to less than high school. Smoking behavior increases the probability of depressive symptoms while drinking behavior reduces it. Each health condition is positively associated with depressive symptoms except for the overweight or obese measure. In Model 5 we interact CRP with race/ethnicity and in Model 6 we interact SNI with race/ethnicity. Research suggests that using point estimates and standard errors to interpret and determine the significance of interactions in nonlinear models is misguided (Ai & Norton, 2003; Mize, 2019). As such we plot the predicted probabilities using estimates from Model 5 in Fig. 1, and the estimates from Model 6 in Fig. 2.

Fig. 1 plots the predicted probabilities of depressive symptoms by race/ethnicity and CRP using the estimates from Model 5 in Table 2.^6^ The y-axis plots the predicted probability of depressive symptoms while the x-axis plots each racial/ethnic group by whether CRP is elevated or not. We indicate CRP levels below the clinical threshold (3.0 mg/dL) with open red circles and elevated CRP levels are marked with solid blue circles. Where CRP levels are unelevated, the predicted probability of depressive symptoms is lower than when CRP levels are elevated within each racial/ethnic group. However, this difference is not significant for the Black population and is marginally significant for the Hispanic population. The difference is statistically significant for the White population, predicting that 10% of those with elevated CRP levels have depressive symptoms compared to just 6% when CRP levels are not elevated.

Fig. 2 is similar to Fig. 1. The difference is that we plot SNI rather than CRP, using Model 6 from Table 2.^7^ Here, an open red circle, a solid blue circle, an open gray square, and a solid black square indicate that SNI is zero, one, two, or three respectively. The pattern is that higher levels of SNI have a lower predicted probability of depressive symptoms relative to lower levels of SNI regardless of racial/ethnic group. However, these differences are only statistically significant among White and Black populations, with the exception of an increase from SNI equals two to SNI equals three. For the Hispanic population, differences are not statistically significant and an increase from zero to three or from one to three in SNI is marginally significant. This is because the Hispanic population only benefits modestly with each increase in SNI relative to the other populations. For example, when SNI equals zero the predicted probability of depressive symptoms among the Hispanic population is 0.14 compared to 0.17 and 0.22 among the White and Black populations, but when SNI equals three these probabilities decline to 0.06, 0.03, and 0.03 respectively. These findings show that increasing social integration decreases the predicted probability of depressive symptoms among the White and Black populations, but not significantly for the Hispanic population.

### 4.1 Sensitivity analyses

We specify regression models using PHQ-9 instead of PHQ-8 (Supplement Table S2) and one finding changed. Using the PHQ-9, when SNI equals two or three, SNI decreases the predicted probability of depressive symptoms relative to an SNI of zero among the Hispanic population, but null associations using the PHQ-8. This finding shows that social integration decreases the likelihood of an affirmative response to the suicidal ideation question among the Hispanic population. We also tested associations using SNI net of marital status (SNI with a range of 0–2) while including marital status as dichotomous independent variable. Our findings remained substantively the same. Furthermore, post-hoc analyses reveal that using three discrete variables finds results that each is negatively associated with depressive symptoms, though future research may want to discern if each integration indicator varies by race/ethnicity. We also include analyses for complete models, like Model 4 of Table 2, without either CRP or SNI (Supplement S3). These results are substantively the same. Lastly, we tested interactions between SNI and CRP (Supplement S3) and found null results.

## 5 Discussion

Established bodies of research suggest that elevated levels of CRP are associated with depressive symptoms and that social integration is negatively associated with depressive symptoms. But research that considers associations between social integration, CRP, and depressive symptoms is nascent and has yet to account for racial/ethnic differences. We bridge this gap by testing these relationships using data nationally representative of White, Black, and Hispanic populations 40 years and older in the United States. This is an important population to consider given that aging populations are at great risk of elevated CRP (Singh & Newman, 2011) and lower levels of social integration (Klinenberg, 2013). We find that elevated levels of CRP are associated with depressive symptoms for the White population, but not for Black or Hispanic populations. We also find that social integration decreases the predicted probability of depressive symptoms, but this relationship is only statistically significant for White and Black populations and not for the Hispanic population. We also find no evidence that social integration influences the relationship between CRP and depressive symptoms.

These results support our first hypothesis that elevated levels of CRP are positively associated with depressive symptoms, but this varies by race/ethnicity as only the White population exhibits a statistically significant association. Though previous research finds the similar results (Case & Stewart, 2014), such work does not incorporate social integration in the analytical model. By including social integration in this analysis, we advance research on the CRP-depressive symptom relationship. Such research often finds that social integration attenuates the relationship between inflammation and health (Uchino et al., 2018). Yet, our findings do not support this.

In naïve models, elevated levels of CRP are strongly associated with depressive symptoms, though controls attenuated the relationship. Post-hoc analysis does not find evidence that a specific control variable attenuates the association between CRP and depressive symptoms, but it is the entirety of controls. These findings are consistent with a review of the association between CRP and depressive symptoms among older adults, where unadjusted models are often significant and adjusted models are not (Smith et al., 2018). Studies that include appropriate confounders find weak associations if any between CRP and depressive symptoms (Horn et al., 2018). Perhaps some control variables influence this association differently by racial/ethnic group given the dynamic processes increasingly apparent in research (Howard & Sparks, 2016). Links between CRP and depressive symptoms vary by physical markers (Jokela et al., 2016), and somatic and non-somatic symptoms (Case & Stewart, 2014). Research that continues to explore these pathways may improve understanding about racial/ethnic variation in the relationship between CRP and depressive symptoms. Even though our findings suggest that this association is not statistically significant for Black and Hispanic populations after accounting for covariates, the predicted probability of depressive symptoms is higher among those with elevated CRP levels regardless of race/ethnicity.

Our findings support our second hypothesis. They show that SNI decreases the predicted probability of depressive symptoms for White and Black populations, though not statistically significant for the Hispanic population. Perhaps cultural and social norms play an important role in why this relationship is not as salient among the Hispanic population (e.g. Desmond & Turley, 2009; Turner & Turner, 2013). For example, familism is linked to positive health outcomes, but it may also lead to high levels of family obligation stress which is negatively associated with health (Molina et al., 2019). Increasing socioeconomic status decreases familial support for Hispanic respondents, but the opposite is true for White and Black respondents (Almeida et al., 2009). Another explanation relies on how the broader social environment may influence health. Social network turnover is unequal by race/ethnicity and across the life course (Cornwell, 2015). This may be especially important for Hispanic populations due to migration patterns. Population density is negatively associated with social integration for White and Hispanic populations, but not the Black population (Jacobson et al., 2018), and the White population is disproportionately concentrated in low density areas perhaps explaining differences in social integration across groups.

Interventions aimed at integration could decrease the predicted probability of depressive symptoms across racial/ethnic groups. Tests of marginal effects (Table S5) based on Model 6 in Table 2 suggest that an increase from one to two in SNI significantly decreases the predicted probability of depressive symptoms from 11.3 percent to 4.5 percent and from 8.2 to 4.8 percent for White and Black populations respectively. Though a similar change in SNI decreases the predicted probability of depressive symptoms among the Hispanic population (10.6–7.1 percent), this difference is not statistically significant while accounting for covariates. Furthermore, estimates suggest that an increase from zero to two in SNI more than halves the predicted probability of depressive symptoms across each racial/ethnic group. Policies aimed at integrating older adults with communities and increasing accessibility to communities may improve mental health outcomes (Vitman et al., 2014).

## 6 Limitations

We conduct the analysis using cross-sectional data, which may not account for reverse causality nor parse bidirectionality. Though extensive research that elaborates on neuropathways (Miller et al., 2009; Miller & Raison, 2016) and uses meta-analyses (Smith et al., 2018; Valkanova et al., 2013) suggests that elevated levels of CRP lead to depressive symptoms, other research argues that depressive symptoms may influence inflammation (Stewart et al., 2009). Relatedly, research finds that social integration precedes depressive symptoms (Uchino et al., 2018), but some argue the opposite (Domènech-Abella et al., 2019). Additionally, health conditions and behaviors are based on self-reports and are subject to recall bias. Furthermore, marginalized populations are subject to disparities in health care (Dovidio et al., 2008) which may lead to fewer diagnoses and influence some control variables of this study.

## 7 Conclusion

Our findings suggest that the association between CRP, social integration, and depressive symptoms varies by racial/ethnic group. Researchers should continue to give attention to subpopulations and incorporate social ties in biodemographic research because factors associated with race/ethnicity and social ties moderate health-related associations (see Bell et al., 2010; Gabriel et al., 2020). Research that examines allostatic load finds that, because such measures are unidimensional, some components of broad measures matter differently across racial/ethnic groups (Howard & Sparks, 2016). Our findings underscore the importance of exploring whether associations vary by subpopulations. Future population-based research should test associations through stratification and interactions to account for differences between subpopulations. This approach to research may help expand scholarly understanding of health determinants and disparities within diverse populations such as the United States and other countries experiencing diversification.

## Ethics statement

Our paper uses publicly available, secondary data, and as such is exempt form ethics review.

## Role of the funding source

This research is supported by funding from the 10.13039/100009633Eunice Kennedy Shriver National Institute of Child Health and Human Development to the Population Research Institute (P2CHD041025) and the 10.13039/100008321Social Science Research Institute at The Pennsylvania State University.

## Declaration of competing interest

The authors have no competing interests to declare.

## Footnotes

1 Hereafter, when we refer to White or Black race, we mean non-Hispanic ethnicity.

2 Researchers operationalize numerous overlapping, but distinct measures of ties in conjunction with inflammation such as: perceived social support (Y. C. Yang, Schorpp, et al., 2014), received social support (Ross et al., 2017), social integration (Loucks et al., 2006), among others.

3 Research finds that the PHQ is robust across racial/ethnic groups (Patel et al., 2019).

4 We omit item 9 of the PHQ-9 and use the first 8 items (PHQ-8) because previous research indicates that item 9 is not an accurate screener question and that the PHQ-8 is more valid (Razykov et al., 2012).

5 In the sociability score, close friends or relatives refers to those that the respondent feels at ease with, can talk about private matters with, and can call on for help when needed.

6 Supplemental Table S4 tests the interactions shown in Fig. 1 and provides calculations of the predicted probabilities.

7 Supplemental Table S5 tests the interaction effects shown in Fig. 2 and provides calculations of the predicted probabilities.
