# Supplementary material for: Racial and ethnic differences in the associations between social integration, C-reactive protein and depressive symptoms
Source: SSM Popul Health. 2020 Sep 4;12:100663. doi: 10.1016/j.ssmph.2020.100663 (PMC7501457; doi:10.1016/j.ssmph.2020.100663)
Supplement: Multimedia component 1 [file mmc1.docx]

Supplement to: Racial and Ethnic Differences in the Associations between Social Integration, C-reactive Protein and Depressive Symptoms

Supplemental Table S1 displays a correlation matrix including all variables from the main analyses of the study. Interestingly, few variables are correlated at 0.2 or greater. The exceptions tend to be variables of the same grouping for example SNI=2 and SNI=3. However, diabetes and hypertension are correlated at 0.21. These associations lead us to believe that there is little or no collinearity among our variables and that including each simultaneously in the analyses helps appropriately model our relationships of interest.

| **Supplemental Table S1**. Correlation Matrix Between Variables Used in Main Analyses. | | | | | | | | | | | | | | | | | | | | | | | | |
| --- | --- | --- | --- | --- | --- | --- | --- | --- | --- | --- | --- | --- | --- | --- | --- | --- | --- | --- | --- | --- | --- | --- | --- | --- |
|  | PHQ-8 | 2 | 3 | 4 | 5 | 6 | 7 | 8 | 9 | 10 | 11 | 12 | 13 | 14 | 15 | 16 | 17 | 18 | 19 | 20 | 21 | 22 | 23 | 24 |
| 2. CRP | 0.05 | 1.00 |  |  |  |  |  |  |  |  |  |  |  |  |  |  |  |  |  |  |  |  |  |  |
| 3. SNI=0 | 0.13 | 0.02 | 1.00 |  |  |  |  |  |  |  |  |  |  |  |  |  |  |  |  |  |  |  |  |  |
| 4. SNI=1 | 0.07 | 0.05 | -0.23 | 1.00 |  |  |  |  |  |  |  |  |  |  |  |  |  |  |  |  |  |  |  |  |
| 5. SNI=2 | -0.07 | -0.03 | -0.27 | -0.53 | 1.00 |  |  |  |  |  |  |  |  |  |  |  |  |  |  |  |  |  |  |  |
| 6. SNI=3 | -0.08 | -0.04 | -0.17 | -0.34 | -0.39 | 1.00 |  |  |  |  |  |  |  |  |  |  |  |  |  |  |  |  |  |  |
| 7. NH White | -0.03 | -0.08 | -0.01 | -0.07 | 0.00 | 0.08 | 1.00 |  |  |  |  |  |  |  |  |  |  |  |  |  |  |  |  |  |
| 8. NH Black | 0.00 | 0.08 | 0.02 | 0.06 | -0.03 | -0.05 | -0.57 | 1.00 |  |  |  |  |  |  |  |  |  |  |  |  |  |  |  |  |
| 9. Hispanic | 0.03 | 0.01 | -0.01 | 0.02 | 0.03 | -0.05 | -0.62 | -0.29 | 1.00 |  |  |  |  |  |  |  |  |  |  |  |  |  |  |  |
| 10. Age 40-54 | 0.07 | -0.04 | 0.04 | 0.02 | 0.00 | -0.05 | -0.09 | 0.02 | 0.09 | 1.00 |  |  |  |  |  |  |  |  |  |  |  |  |  |  |
| 11. Age 55-64 | 0.02 | 0.04 | -0.01 | 0.01 | -0.01 | 0.01 | -0.11 | 0.07 | 0.05 | -0.45 | 1.00 |  |  |  |  |  |  |  |  |  |  |  |  |  |
| 12. Age 65-74 | -0.06 | 0.01 | -0.02 | -0.01 | 0.00 | 0.03 | 0.02 | 0.01 | -0.04 | -0.40 | -0.28 | 1.00 |  |  |  |  |  |  |  |  |  |  |  |  |
| 13. Age 75+ | -0.05 | 0.00 | -0.01 | -0.03 | 0.02 | 0.02 | 0.21 | -0.12 | -0.13 | -0.36 | -0.25 | -0.23 | 1.00 |  |  |  |  |  |  |  |  |  |  |  |
| 14. Less than HS | 0.09 | 0.05 | 0.09 | 0.08 | -0.05 | -0.10 | -0.27 | 0.03 | 0.29 | -0.09 | -0.01 | 0.05 | 0.07 | 1.00 |  |  |  |  |  |  |  |  |  |  |
| 15. High School | -0.01 | 0.01 | -0.02 | 0.00 | 0.01 | 0.01 | 0.13 | 0.00 | -0.16 | 0.04 | -0.01 | -0.03 | -0.01 | -0.65 | 1.00 |  |  |  |  |  |  |  |  |  |
| 16. College | -0.09 | -0.07 | -0.07 | -0.09 | 0.05 | 0.10 | 0.14 | -0.04 | -0.13 | 0.04 | 0.02 | -0.03 | -0.06 | -0.33 | -0.50 | 1.00 |  |  |  |  |  |  |  |  |
| 17. Non-smoker | -0.07 | -0.04 | -0.08 | -0.08 | 0.05 | 0.09 | -0.08 | 0.01 | 0.08 | 0.03 | -0.01 | -0.03 | 0.01 | -0.05 | -0.03 | 0.09 | 1.00 |  |  |  |  |  |  |  |
| 18. Current Smoker | 0.13 | 0.06 | 0.15 | 0.12 | -0.09 | -0.14 | -0.01 | 0.07 | -0.05 | 0.17 | 0.02 | -0.08 | -0.16 | 0.09 | 0.03 | -0.14 | -0.47 | 1.00 |  |  |  |  |  |  |
| 19. Former Smoker | -0.03 | -0.01 | -0.04 | -0.02 | 0.02 | 0.02 | 0.10 | -0.07 | -0.05 | -0.18 | 0.00 | 0.10 | 0.12 | -0.03 | 0.01 | 0.02 | -0.67 | -0.34 | 1.00 |  |  |  |  |  |
| 20. Never Drink | 0.05 | 0.06 | -0.01 | 0.03 | -0.01 | -0.01 | -0.05 | 0.08 | -0.02 | -0.12 | 0.02 | 0.05 | 0.07 | 0.13 | -0.03 | -0.12 | -0.08 | -0.01 | 0.09 | 1.00 |  |  |  |  |
| 21. Drink | 0.02 | 0.02 | 0.04 | -0.01 | -0.02 | 0.00 | -0.03 | -0.03 | 0.06 | 0.10 | 0.02 | -0.05 | -0.11 | -0.08 | 0.06 | 0.01 | 0.02 | 0.03 | -0.04 | -0.39 | 1.00 |  |  |  |
| 22. Heavy Drinker | -0.06 | -0.07 | -0.02 | -0.02 | 0.03 | 0.01 | 0.07 | -0.05 | -0.03 | 0.01 | -0.04 | -0.01 | 0.04 | -0.05 | -0.03 | 0.09 | 0.06 | -0.02 | -0.04 | -0.53 | -0.58 | 1.00 |  |  |
| 23. Diabetes | 0.06 | 0.08 | 0.01 | 0.01 | 0.01 | -0.03 | -0.13 | 0.11 | 0.05 | -0.15 | 0.06 | 0.10 | 0.01 | 0.11 | -0.03 | -0.09 | 0.00 | -0.05 | 0.04 | 0.12 | -0.04 | -0.07 | 1.00 |  |
| 24. Hypertension | 0.06 | 0.10 | -0.01 | 0.03 | -0.01 | -0.01 | -0.01 | 0.12 | -0.10 | -0.25 | 0.06 | 0.14 | 0.12 | 0.03 | 0.01 | -0.05 | 0.00 | -0.07 | 0.06 | 0.07 | -0.05 | -0.01 | 0.21 | 1.00 |
| BMI 25+ | 0.02 | 0.19 | -0.04 | 0.01 | 0.01 | 0.01 | -0.08 | 0.02 | 0.08 | 0.00 | 0.05 | 0.02 | -0.08 | 0.00 | 0.04 | -0.05 | 0.05 | -0.14 | 0.07 | 0.04 | 0.06 | -0.09 | 0.12 | 0.14 |
| Note: The matrix is symmetrical. The first column compares PHQ-8 with all other variables so it is not included as a row, the opposite is true for BMI. | | | | | | | | | | | | | | | | | | | | | | | | |

Supplemental Table S2 is the same analysis as that of Table 2 in the manuscript; except that the outcome variable is the PHQ-9 rather than the PHQ-8. Overall, this model is quite similar to that in the manuscript. The major difference is that differences between the Hispanic and White or Black populations in SNI no longer persist in this model. This suggests that the omitted question from PHQ-8 regarding suicidal ideation has an important influence on whether or not the Hispanic population meets the depressive symptom threshold and responses to that question may be disproportionately correlated with social integration among this population. Social integration may be especially protective of this ideation for Hispanic respondents relative to other groups. Future analyses may consider how social integration influences each item of depression questionnaires like the PHQ-9.

| **Supplemental Table S2.** Weighted logistic regression models for Depression as PHQ-9, CRP, and SNI. Controlling for Sociodemographic Characteristics and Health Behaviors for U.S. Adults 40 and older, stratified by race/ethnicity, NHANES 2005-2008 (n=5,634). | | | | | | | | | | | | |
| --- | --- | --- | --- | --- | --- | --- | --- | --- | --- | --- | --- | --- |
|  | Model 1 | | Model 2 | | Model 3 | | Model 4 | | Model 5 | | Model 6 | |
|  | OR | SE | OR | SE | OR | SE | OR | SE | OR | SE | OR | SE |
| Clinical CRP | 1.557*** | (0.182) |  |  | 1.483** | (0.170) | 1.174 | (0.146) | 1.236 | (0.185) | 1.173 | (0.146) |
| Social Network Index |  |  |  |  |  |  |  |  |  |  |  |  |
| SNI=1 |  |  | 0.601*** | (0.080) | 0.602*** | (0.081) | 0.628** | (0.092) | 0.628** | (0.093) | 0.740 | (0.142) |
| SNI=2 |  |  | 0.271*** | (0.040) | 0.275*** | (0.041) | 0.338*** | (0.051) | 0.338*** | (0.052) | 0.336*** | (0.066) |
| SNI=3 |  |  | 0.168*** | (0.031) | 0.171*** | (0.031) | 0.242*** | (0.051) | 0.242*** | (0.051) | 0.251*** | (0.068) |
| Race/Ethnicity |  |  |  |  |  |  |  |  |  |  |  |  |
| Black | 1.290 | (0.240) | 1.184 | (0.192) | 1.135 | (0.188) | 0.869 | (0.144) | 0.963 | (0.229) | 1.464 | (0.482) |
| Hispanic | 1.744** | (0.286) | 1.686** | (0.279) | 1.660** | (0.271) | 1.413* | (0.215) | 1.547* | (0.244) | 1.339 | (0.515) |
| Age Group |  |  |  |  |  |  |  |  |  |  |  |  |
| Age 55-64 |  |  |  |  |  |  | 0.664** | (0.085) | 0.666** | (0.085) | 0.666** | (0.085) |
| Age 65-74 |  |  |  |  |  |  | 0.473*** | (0.065) | 0.473*** | (0.065) | 0.480*** | (0.067) |
| Age 75+ |  |  |  |  |  |  | 0.640* | (0.122) | 0.639* | (0.122) | 0.649* | (0.123) |
| Male |  |  |  |  |  |  | 0.699** | (0.090) | 0.699** | (0.090) | 0.696** | (0.090) |
| Educational Attainment |  |  |  |  |  |  |  |  |  |  |  |  |
| HS Graduate |  |  |  |  |  |  | 0.753 | (0.133) | 0.753 | (0.133) | 0.756 | (0.134) |
| College Graduate |  |  |  |  |  |  | 0.393*** | (0.092) | 0.394*** | (0.093) | 0.398*** | (0.091) |
| Health Behaviors |  |  |  |  |  |  |  |  |  |  |  |  |
| Current Smoker |  |  |  |  |  |  | 1.867** | (0.316) | 1.859** | (0.314) | 1.865** | (0.318) |
| Former Smoker |  |  |  |  |  |  | 1.037 | (0.147) | 1.038 | (0.147) | 1.040 | (0.148) |
| Mild Drink |  |  |  |  |  |  | 0.837 | (0.091) | 0.836 | (0.091) | 0.836 | (0.088) |
| Drinker |  |  |  |  |  |  | 0.688*** | (0.066) | 0.690*** | (0.066) | 0.681*** | (0.064) |
| Health Conditions |  |  |  |  |  |  |  |  |  |  |  |  |
| Diabetes |  |  |  |  |  |  | 1.589* | (0.267) | 1.588* | (0.266) | 1.585* | (0.270) |
| Hypertension |  |  |  |  |  |  | 1.796*** | (0.282) | 1.796*** | (0.282) | 1.790** | (0.284) |
| Overweight/Obese |  |  |  |  |  |  | 1.104 | (0.159) | 1.101 | (0.158) | 1.103 | (0.159) |
| Interactions |  |  |  |  |  |  |  |  |  |  |  |  |
| Black x SNI=1 |  |  |  |  |  |  |  |  |  |  | 0.428** | (0.127) |
| Hispanic x SNI=1 |  |  |  |  |  |  |  |  |  |  | 0.693 | (0.248) |
| Black x SNI=2 |  |  |  |  |  |  |  |  |  |  | 0.679 | (0.284) |
| Hispanic x SNI=2 |  |  |  |  |  |  |  |  |  |  | 1.552 | (0.717) |
| Black x SNI=3 |  |  |  |  |  |  |  |  |  |  | 0.499 | (0.330) |
| Hispanic x SNI=3 |  |  |  |  |  |  |  |  |  |  | 1.523 | (0.799) |
| Black x CRP |  |  |  |  |  |  |  |  | 0.817 | (0.206) |  |  |
| Hispanic x CRP |  |  |  |  |  |  |  |  | 0.821 | (0.213) |  |  |
| Constant | 0.074*** | (0.011) | 0.225*** | (0.037) | 0.190*** | (0.035) | 0.272*** | (0.074) | 0.266*** | (0.074) | 0.253*** | (0.077) |
| Standard errors in parentheses. *** p<0.001, ** p<0.01, * p<0.05, † p<0.1 | | | | | | | | | | | | |
| Note: Reference group for SNI is zero and race/ethnicity is White. Reference groups for age group is 40-54, for educational attainment is less than high school, reference group for health behaviors and conditions are no condition, and no behavior. | | | | | | | | | | | | |

Supplemental Table S3 presents full models for CRP and SNI while excluding the other. Additionally, it also includes an interaction model between CRP and SNI. Model 1 and Model 2 reflect comparable results to Model 4 of the main analyses. In Model 1, after accounting for control variables, the relationship between CRP and depressive symptoms is attenuated. In Model 2, SNI is still negatively associated with depressive symptoms even when CRP is not included in the model. In Model 3 we include an interaction term between CRP and SNI. These results do not reveal new associations. The predicted probability of depressive symptoms is negatively associated with SNI, and positively associated with CRP. However, there appears to be no relationship between SNI and CRP.

| **Supplemental Table S3**. Predicted Probability of Depressive Symptoms including all control measures with and without CRP and SNI and interaction terms between CRP and SNI (N=5,634) | | | | | | |
| --- | --- | --- | --- | --- | --- | --- |
|  | Model 1 | | Model 2 | | Model 3 | |
|  | OR | SE | OR | SE | OR | SE |
| Elevated CRP | 1.247 | (0.161) |  |  | 0.883 | (0.261) |
| Social Network Index |  |  |  |  |  |  |
| SNI=1 |  |  | 0.600** | (0.106) | 0.488* | (0.126) |
| SNI=2 |  |  | 0.313*** | (0.059) | 0.281*** | (0.063) |
| SNI=3 |  |  | 0.223*** | (0.054) | 0.145*** | (0.041) |
| Race/Ethnicity |  |  |  |  |  |  |
| Black | 1.002 | (0.166) | 0.949 | (0.150) | 0.935 | (0.149) |
| Hispanic | 1.195 | (0.183) | 1.189 | (0.195) | 1.190 | (0.196) |
| Age Group |  |  |  |  |  |  |
| Age 55-64 | 0.634*** | (0.071) | 0.651*** | (0.074) | 0.655** | (0.075) |
| Age 65-74 | 0.421*** | (0.070) | 0.416*** | (0.071) | 0.419*** | (0.069) |
| Age 75+ | 0.466*** | (0.090) | 0.472*** | (0.090) | 0.474*** | (0.091) |
| Male | 0.602*** | (0.074) | 0.604*** | (0.082) | 0.624** | (0.082) |
| Educational Attainment |  |  |  |  |  |  |
| HS Graduate | 0.683† | (0.129) | 0.750 | (0.143) | 0.755 | (0.144) |
| College Graduate | 0.311*** | (0.088) | 0.364** | (0.103) | 0.373** | (0.104) |
| Health Behaviors |  |  |  |  |  |  |
| Current Smoker | 2.557*** | (0.391) | 2.038*** | (0.357) | 1.964*** | (0.353) |
| Former Smoker | 1.294 | (0.230) | 1.242 | (0.230) | 1.235 | (0.229) |
| Drinker | 0.933 | (0.098) | 0.919 | (0.096) | 0.931 | (0.098) |
| Heavy Drinker | 0.673** | (0.083) | 0.665** | (0.080) | 0.677** | (0.082) |
| Health Conditions |  |  |  |  |  |  |
| Diabetes | 1.553* | (0.295) | 1.546* | (0.298) | 1.526* | (0.299) |
| Hypertension | 1.772** | (0.332) | 1.778** | (0.334) | 1.754** | (0.334) |
| Overweight/Obese | 1.096 | (0.199) | 1.200 | (0.214) | 1.129 | (0.198) |
| Interactions |  |  |  |  |  |  |
| CRP x SNI=1 |  |  |  |  | 1.556 | (0.585) |
| CRP x SNI=2 |  |  |  |  | 1.271 | (0.415) |
| CRP x SNI=3 |  |  |  |  | 2.411† | (1.061) |
| Constant | 0.103*** | (0.025) | 0.239*** | (0.069) | 0.262*** | (0.084) |
| Standard errors in parentheses. *** p<0.001, ** p<0.01, * p<0.05, † p<0.1 | | | | | | |
| Note: Reference group for SNI is zero and race/ethnicity is White. Reference groups for age group is 40-54, for educational attainment is less than high school, reference group for health behaviors and conditions are no condition, and no behavior. | | | | | | |

Supplemental Table S4 provides the marginal effects of CRP (first difference) and the equality of those marginal effects (second differences) of CRP across each racial/ethnic group. The first two columns indicate the predicted probability of depressive symptoms when CRP is below 3.0 mg/dL (CRP=0) or above 3.0 mg/dL (CRP=1). The third column is the difference in predicted probability of depression between when CRP equals zero and when CRP equals one. For example, when CRP equals zero for the Hispanic population, the predicted probability of depressive symptoms is 0.067, but when CRP equals one, the predicted probability increases to 0.105. That difference is marginally significant among the Hispanic population. The difference across CRP is not statistically significant for the Black population, but it is for the White population. Tests for second differences reveal that the difference in marginal effects between groups is not statistically significant. In other words, the interaction term between CRP and race/ethnicity is not statistically significant.

| **Supplemental Table S4**. Marginal Effects and Second Differences of Race/Ethnicity on Predicted Probability of Depressive Symptoms by CRP. | | | |
| --- | --- | --- | --- |
|  | CRP=0 | CRP=1 | AME |
| White | 0.056 | 0.097 | 0.040* |
| Black | 0.068 | 0.089 | 0.021 |
| Hispanic | 0.067 | 0.105 | 0.038† |
| Significance levels: * p<0.05, † p<0.1. | | | |
| Note: AME are average marginal effects, the difference between CRP equals zero and one for each race/ethnicity. CRP = 0 represents C-reactive protein levels that are below 3.0 mg/dL and CRP = 1 indicate levels above 3.0 mg/dL. Estimates include all coefficients from Model 5 in Table 2. | | | |

Supplemental Table S5 shows the marginal effects and second differences of the interaction from Model 6 in Table 2 of the manuscript. In the first four columns we show the predicted probabilities of depression for each racial/ethnic group for each SNI value. In the remaining six columns we calculate the difference between each SNI value and test for statistical significance within each racial/ethnic group. We use subscript characters for differences between race/ethnic groups. As an example, in the second row, which references the Black population, when SNI equals one, the predicted probability of depressive symptoms is 0.082 or about 8%. When SNI equals three the predicted probability is 0.027 or about 3%. In the column labeled SNI 1-3 we see that the difference is 0.055 and the asterisk indicates a statistically significant difference between the predicted probability of depressive symptoms when SNI equals one and when SNI equals three for the Black population. Overall, we see that there are significant differences between every comparison across SNI for the Black population aside from the comparison between when SNI equals two versus three. For the White population, there is a similar trend, though the difference from zero to one is marginally significant. The pattern is different for the Hispanic population where no differences are statistically significant and only differences between zero and three or one and three are marginally significant. We test for second differences and find that the difference between zero and one, zero and two, as well as zero and three are statistically different comparing the Black population to the Hispanic population. These formal tests reinforce the findings of Figure 2 presented in the manuscript and show evidence of a statistically significant interaction between race/ethnicity and SNI in this logistic regression model.

| **Supplemental Table S5.** Marginal Effects and Second Differences of Race/Ethnicity on Predicted Probability of Depressive Symptoms by SNI. | | | | | | | | | | | |
| --- | --- | --- | --- | --- | --- | --- | --- | --- | --- | --- | --- |
|  |  |  |  |  |  | | | | | |  |
|  | SNI=0 | SNI=1 | SNI=2 | SNI=3 | SNI 0-1 | SNI 0-2 | SNI 0-3 | SNI 1-2 | SNI 1-3 | SNI 2-3 |  |
| White | 0.167 | 0.113 | 0.045 | 0.027 | -0.054† | -0.122* | -0.140* | -0.068* | -0.086* | -0.018 |  |
| Black | 0.221 | 0.082 | 0.048 | 0.027 | -0.139_c_ | -0.173*_c_ | -0.194*_c_ | -0.034* | -0.055* | -0.021 |  |
| Hispanic | 0.135 | 0.106 | 0.071 | 0.060 | -0.029_b_ | -0.064_b_ | -0.075†_b_ | -0.035 | -0.046† | -0.011 |  |
| Significance levels: * p<0.05, † p<0.1. | | | | | | | | | | | |
| Note: Estimates include all coefficients from Model 6 in Table 2. Subscript characters reveal whether the comparison is different across racial/ethnic groups. a, b, c, refer to White, Black, or Hispanic respectively. | | | | | | | | | | | |
